# MOF-Based Biosensors for the Detection of Carcinoembryonic Antigen: A Concise Review

**DOI:** 10.3390/molecules28165970

**Published:** 2023-08-09

**Authors:** May R. Ibrahim, Yaser E. Greish

**Affiliations:** Department of Chemistry, United Arab Emirates University, Al Ain P.O. Box 15551, United Arab Emirates

**Keywords:** biosensors, metal–organic frameworks, carcinoembryonic antigen

## Abstract

Cancer has been considered one of the most serious diseases in recent decades. Early diagnosis of cancer is a crucial step for expedited treatment. Ideally, detection of cancer biomarkers, which are usually elevated because of cancer, is the most straightforward approach to detecting cancer. Among these biomarkers, the carcinoembryonic antigen (CEA) is considered one of the most important tumor markers for colorectal cancer. The CEA has also been recognized as a biomarker for other types of cancers, including breast, gastric, ovarian, pancreatic, and lung cancers. Typically, conventional CEA testing depends on immunoassay approaches, which are known to be complex, highly expensive, and time consuming. Accordingly, various types of biosensors have been designed for the detection of cancer biomarkers. The main prerequisites of these biosensors are high sensitivity, fast response, and low cost. Many nanostructures have been involved in the design of biosensors, such as nanoparticles of certain metals and metal oxides that are further functionalized to contribute to the sensing of the biomarkers. Alternatively, metal organic frameworks (MOFs), which are extended crystalline structures comprising metal clusters surrounded by organic linkers, have been shown to be highly promising for the development of biosensors. The 3D structure of MOFs results in a combination of high surface area and high interconnected porosity, which are believed to facilitate their function in the design of a biosensor. This review briefly classifies and describes MOF-based biosensor trials that have been published recently for the aim of detecting CEA.

## 1. Background

Globally, cancer has been recorded as one of the primary etiological causes of death. Cancer is described as an abnormal and uncontrolled cell growth resulting from an accumulation of specific genetic and epigenetic defects. The underlying origins of cancer are either environmental or hereditary [1]. Classically, physicians and scientists rely on methods such as biopsy, cross-sectional imaging (CT scan), and endoscopy techniques to discover cancer. However, these methods are invasive and expensive for most patients. Accordingly, many efforts have been made to explore innovative approaches for the diagnosis of cancer. After the discovery of cancer biomarkers, cancer detection becomes less invasive, as these biomarkers can be detected in body fluids.

Biomarkers can be defined as specific macromolecules that are usually found in bodily fluids or tissues as an indication of the development of cancer. It has been found that early detection of cancer biomarkers has a great impact on controlling the disease in the term of treatment or even surgical interference in many incidents [2]. Biomarkers can be whole cells, biomolecules, or genetic materials. There are up to 160 biomarkers that have been proven to detect different types of cancer [3]. One of the most common biomarkers is carcinoembryonic antigen (CEA, also known as CEACAM5 or CD66e). CEA overexpression is related to colon, lung, ovarian, and breast cancer [4]. This review outlines the various methods of the detection of CEA, with special emphasis on the use of MOF nanomaterials in this regard.

### 1.1. Structure and Function of CEA

**Figure 1:**
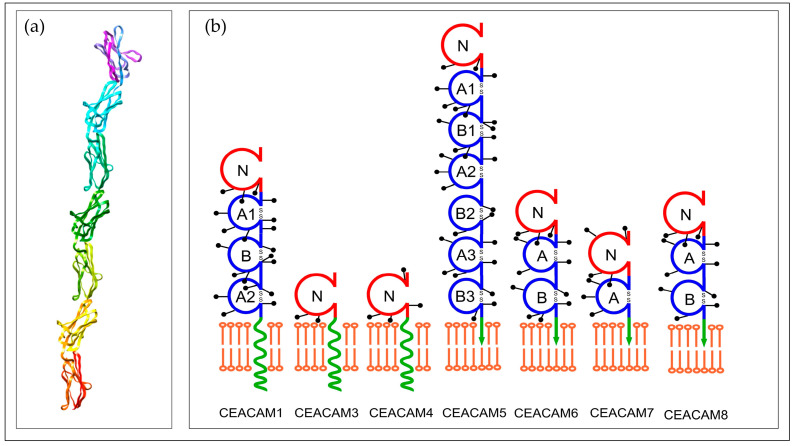
Three-dimensional representation of CEACAM5 (figure modified using Chimera 1.17 software from https://www.rcsb.org/structure/1e07 (accessed on 22 July 2023)) (**a**) and schematic representation for the CEA family (**b**) (graph modified from http://www.carcinoembryonic-antigen.de (accessed on 22 July 2023) illustrating different chains (A1, A2, A3, B1, B2, B3 etc) in different members of CEA proteins family).

CEA is a glycosylated protein of a high molecular weight (180–200 KDa), as shown in Figure 1a. It is involved in the cell adhesion and recognition mechanisms (PDB entry 1e07) [5]. Gold and Freedman were the pioneers in describing CEA sixty years ago. They realized that CEA was related to human colon cancer tissue extracts and later to digestive system cancer [4]. CEA has immunoglobulin-like structural features and many glycosylation modification sites [6]. Particularly, CEA belongs to the immunoglobulin superfamily of cell adhesion molecules (IgCAMs). IgCAMs are highly glycosylated surface proteins whose function is fundamental in cell–cell adhesion [2].

The function of CEA in a normal individual is not well understood. Naturally, CEA is secreted in the fetal intestinal tissue, and its serum level can also be elevated in nonmalignant diseases such as inflammatory bowel disease. In the tissues of adults, CEA-related cell surface molecules are expressed primarily in different epithelia, vessel endothelia, and hematopoietic cells [7]. Conversely, in healthy adults, CEA serum level is usually found in low concentrations. As a result, its abnormal elevation is associated with gastric, breast, ovarian, pancreatic, and most importantly colorectal cancer [8,9].

The family of the CEA-related cell adhesion molecules (CEACAM), which are expressed from the CEA gene family, consists of a single N-terminal domain and a maximum of six disulfide-linked internal domains. The group contains 12 proteins (CEACAM1, 3–8, 16, 18–21), as shown in Figure 1b [10]. The extracellular domains of the CEACAM group’s function are homophilic and heterophilic cellular adhesion molecules or receptors.

**Figure 2:**
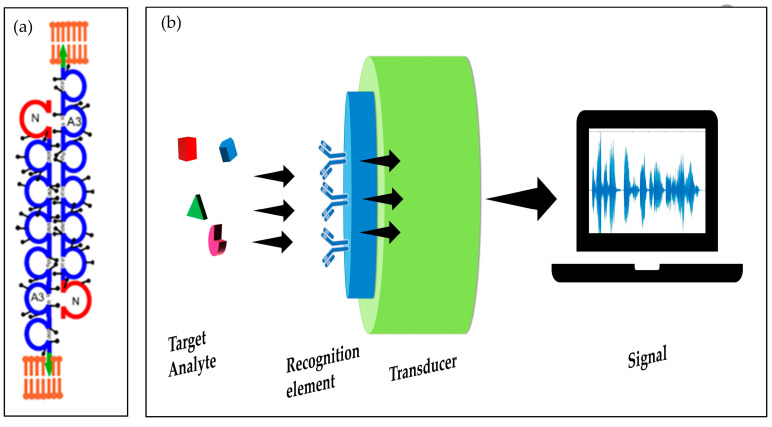
(**a**) shows homotypic binding between two CEA molecules. (CEA structures are modified from the CEA Site http://cea.klinikum.uni-muenchen.de (accessed on 22 July 2023)). (**b**) Graphical illustration of biosensors parts.

It was found that the ability of CEA for cell adhesion on the surface of epithelial cells originates from these properties. This leads to tumor growth through the development of CEA–CEA bridges between tumor cells or tumor–normal cells, as shown in Figure 2a.

The early detection of the carcinoembryonic antigen (CEA) biomarker is of significant importance for several reasons, particularly in the context of cancer diagnosis and management. Detecting cancer at an early stage greatly improves the chances of successful treatment and long-term survival. Regular CEA testing, especially in high-risk individuals or those with a family history of cancer, can aid in identifying potential malignancies at their earliest stages [11]. For patients already diagnosed with cancer, monitoring CEA levels over time can help track the progression of the disease and response to treatment. A decline in CEA levels after treatment may indicate a positive response, while a rise in levels might suggest disease recurrence or metastasis, prompting further investigation and timely intervention. CEA levels are typically measured through a simple blood draw. The blood sample is then sent to a laboratory where specialized assays are used to quantify the CEA concentration. Apart from blood, CEA can also be detected in other body fluids, such as urine [12]. CEA can be found in urine, particularly in cases of certain cancers like bladder cancer and colorectal cancer with metastases to the urinary tract. However, urine CEA testing is not as common as blood testing.

### 1.2. Detection of CEA Biomarker: Conventional Methods vs. Biosensors

ELISA (enzyme-linked immunosorbent assay) was the utmost used method for CEA detection [8]. Numerous approaches for the detection of the CEA biomarker have been explored. These include radioimmunoassay (RIA) and immunoradiometric assay (IRMA), reverse transcriptase polymerase chain reaction (RT-PCR) [1], capillary electrophoresis, and chromatographic analysis [13]. In spite of their reliable detection, most established assays are still relatively considered as complex, time consuming, and expensive. In addition, most of these techniques require the availability of professionally skilled personnel to carry out their protocols. Hence, replacing these ways with other assays that are highly sensitive, highly selective, rapid, and cost-effective for the detection of CEA is required as an approach to the detection of human cancer. With the development of biosensor technology, biosensors have attracted great attention as a tool for the analysis and diagnosis of cancer biomarkers in a simple, selective, and low-cost manner.

A typical biosensor usually consists of three main parts: firstly, a biosensing (or biorecognition) component for the selective recognition; secondly, a transducer and a signal processing unit; while the third part is the detector. The transducer can transfer the signals produced from the biorecognition part in different methods including optical, piezoelectric, electrochemical, magnetic, and thermal [14]. Several studies have reported the fabrications of CEA biosensors with good selectivity and sensitivity [8] (as shown in Figure 2b).

The recognition parts of CEA biosensors were frequently made up of a broad variety of nanomaterials, such as gold nanoparticles [15], graphene quantum dots [16], and molecular imprinted polymers (MIPs) [17]. The common objective of these sensors has been the development of highly sensitive and highly selective approaches that result in the achievement of satisfying results, as compared to conventional methods [18].

### 1.3. Why MOFs Are Used for Biosensors

**Figure 3:**
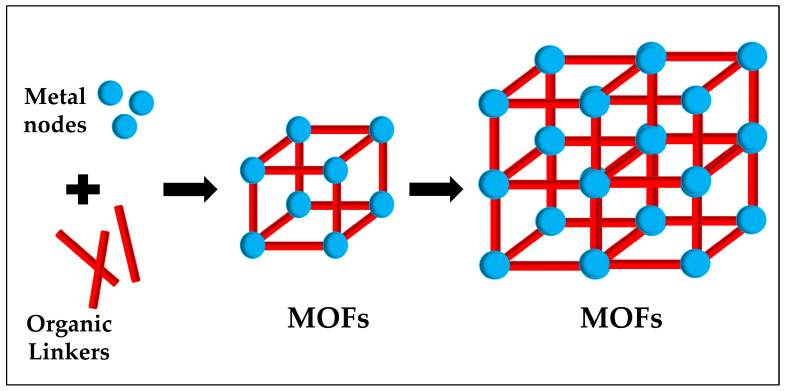
Scheme for the preparation of an MOF.

In the same context, MOFs have recently been recognized as potential materials for enhancing the sensitivity and selectivity of biosensors for the detection of cancer biomarkers. A typical MOF structure essentially consists of metal nodes and organic linkers known as struts (Figure 3). The arrangement of metal nodes and struts in an orderly fashion in their geometrical configuration results in uniform porous crystalline structures of MOFs with outstanding tunable porosity [19]. The hierarchical structure of MOFs provides unique features, such as high surface area that facilitates the interaction with more analytes. This huge surface area allows for more efficient immobilization of bioreceptors, such as enzymes, antibodies, or DNA probes, onto its surface. These bioreceptors are responsible for selectively interacting with the target analyte (can be CEA or other analyte). By increasing the surface area available for immobilization, more bioreceptors can be attached, leading to higher sensitivity and better detection of the target analyte [20].

In addition, a tunable porosity enables the capture of biomarkers with diverse structure and reactivity. Moreover, The functional groups present in MOFs (metal–organic frameworks) can be utilized to immobilize proteins through various chemical interactions such as physical adsorption, covalent bonding, biomimetic mineralization, etc.

Moreover, MOFs can be fabricated in various dynamic geometrical shapes (0D, 1D, 2D, 3D), hence providing more options for capturing more analytes within the framework [20]. Additionally, the facile synthesis of multifunctional MOFs has extended their applications in the fields of energy storage, wastewater treatment, photovoltaics, and biosensing [18,21].

**Figure 4:**
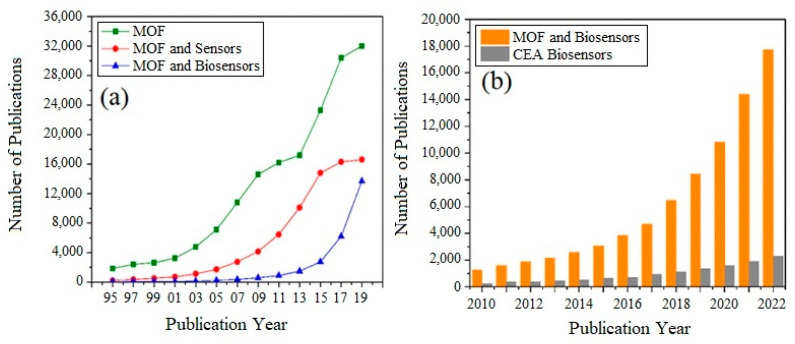
(**a**) Number of publications of sensor-based MOFs in the period 1995–2019 based on Google Scholar, (**b**) articles published from the year 2010 to 2022 with the keyword “MOF and Biosensors” and “Cancer embryonic Antigen Biosensors” obtained and compiled from the Google Scholar database on the 7 February 2023.

The first attempts to design biosensors were carried out in 2008 [22]. A significant exponential increase in interest in biosensors in general and MOF-based biosensors in particular has been observed since 2008, as shown in Figure 4. This was also accompanied with a consistent increase in the development of MOF nanostructures with various functionalities, morphologies, and capabilities. Moreover, hybrid nanostructures involving MOFs and other nanomaterials, such as gold nanoparticles (Au NPs), graphene oxide, carbon nanotubes (CNTs) have shown promising results as composite biosensors. This is attributed to the enhanced optical or electrochemical signals generated during the sensing process [23,24].

**Figure 5:**
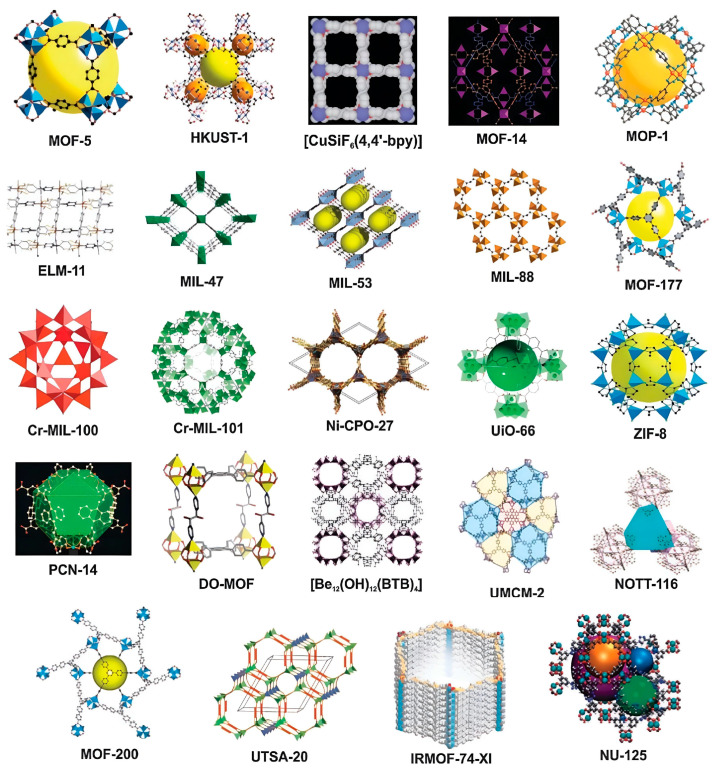
Illustration of nonporous structures of different MOFs members synthesized by different research groups. (Several names are assigned to the synthetized MOFs by different groups of research) Reproduced from Ref. [19], with permission from the Royal Society of Chemistry.

**Figure 6:**
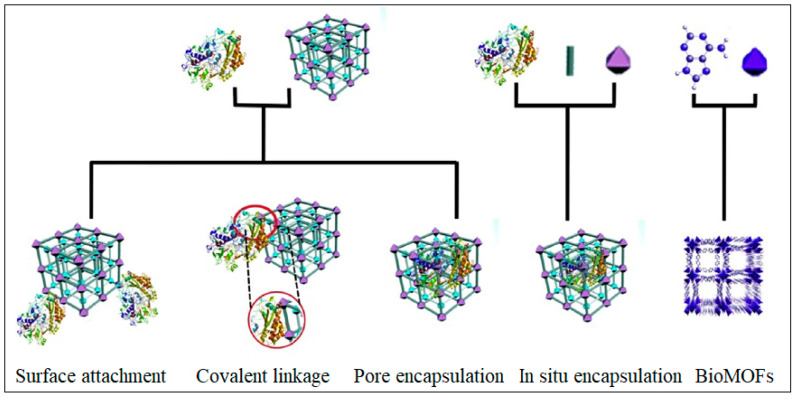
Schematic representation of different immobilization methods. Reproduced from Ref. [26] with permission from Elsevier.

Interestingly, the unique surface functionality and the highly interconnected porosity of MOF nanostructures can also serve as sites for the immobilization of bioactive molecules that can help in the biosensing process, such as oligonucleotides, antibodies, and enzymes [25]. Figure 5 shows typical MOF nanostructures that have been explored for the development of biosensors and as drug delivery vehicles for cancer treatment. Immobilization of these bioactive molecules usually takes place through any of the approaches shown in Figure 6. The choice of immobilization approach depends to a large extent on the size of the molecules as well as the MOF’s surface functionalities and the average pore size [26,27]. Moreover, other materials such as polymers, carbon materials, and metal nanoparticles could be considered in the design of the biosensor in order to contribute to the sensing mechanism. Recently, abundant studies have described the biosensing applications of MOFs either as a stand-alone candidate or combined with other structures, which have shown promising potential applications [24].

MOF nanostructures have been prepared by different methods, including hydrothermal [28], solvothermal [29], ultrasonication [30], electrochemical [31], mechanochemical [32], and microwave-assisted heating [33]. Facile methods of synthesizing MOFs provide the opportunity to optimize the composition and crystalline arrangement of metal nodes and struts in order to obtain desired porosity, surface area, and reactivity for an application of interest [19]. In the following sections, examples of the most recent advances in the design and fabrication of MOF-based biosensors for the detection of CEA will be illustrated.

## 2. Progress in MOF-Based Biosensors for the Detection of CEA

Recently, many research papers have described the design of MOF-based biosensors for CEA detection via different routes, namely electrochemical, optical, photoelectrochemical, etc. The application of MOF-based biosensors for the detection of CEA using these approaches is explained in the following sections.

### 2.1. Electrochemical Approach

Electrochemical detection mainly depends on the transduction of the chemical reactions to electrical signal (e.g., current, voltage, impedance, etc.). By incorporating this kind of detection in biosensors, biochemical reactions such as the enzyme substrate reaction and antigen–antibody can be translated to electrical signals. Many electrochemical sensors have proven their efficiency and have been commercialized. In these biosensors, the working electrode is a key component, which is employed as a solid support for the of immobilization of biomolecules (enzyme, antibody, and nucleic acid) and electron movement [34,35]. The electrochemical cell used in these sensors is composed of three electrodes submerged in an electrolyte of the required analytes. One of the electrodes is a reference electrode (such as Ag/AgCl or Hg/Hg_2_Cl_2_), which is used to maintain a constant potential when compared to the working electrode. The other electrode is known as the working electrode, where the reaction of interest takes place. The third electrode, known as the auxiliary electrode, is composed of an inert conducting substance (e.g., platinum or graphite). Considerable interest in the development MOF-based biosensors has been shown, which is attributed to the high chemical stability of MOFs. This results in an excellent electrochemical biosensor performance, where the MOF nanostructure is immobilized on the working electrode [36,37]. Many electrochemical analysis techniques have been used in these biosensors, such as cyclic voltammetry (CV), differential pulse voltammetry (DPV), square wave voltammetry (SWV), and electrochemical impedimetric signal (EIS). Examples of MOF-based biosensors are described in the following sections.

Zhang et al. presented a CEA electrochemical biosensor that was based on the modification of a glassy carbon electrode (GCE) using polyaniline nanofibers. Polyaniline nanofibers provided high surface area and good electrical conductivity. The modified GCE was investigated for the simultaneous determination of the cancer biomarkers CEA and alpha-fetoprotein (AFP) by using particles of MOFs prepared from Pb(II) or Cd(II). The MOFs nanoparticles were labeled by secondary antibodies for both biomarkers to form a sandwich assay. The respective detection limits were 0.03 pg/mL and 0.1 pg/mL, respectively [38].

A similar sandwich-type electrochemical biosensor was proposed by Li’s group [39]. In their work, Ce MOF was synthetized and loaded with silver nanoparticles (Ag NPs) and horseradish peroxidase to catalyze H_2_O_2_, hence amplifying the current signal. This MOF-based assembly was then coated with hyaluronic acid. The designed immune sensor further used Au NPs to enhance the ability of attaching the antibody based on the high electrical conductivity of the Au NPs. The immune sensor’s assembly was tested for the detection of CEA and showed a limit of detection (LOD) of 0.2 pg/mL. Moreover, the proposed immune sensor was found to possess high reproducibility, selectivity, and stability [39].

**Figure 7:**
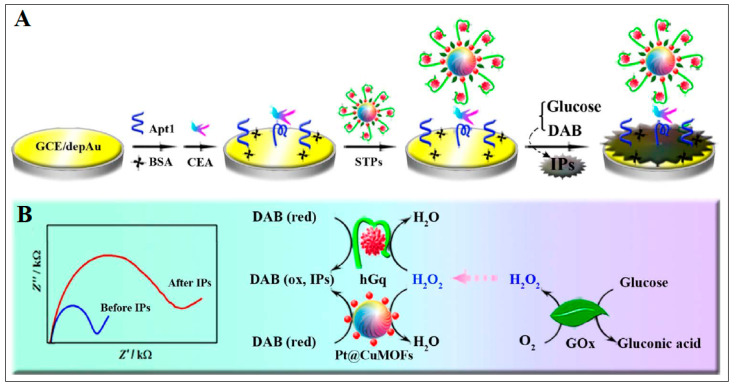
Example for Cu MOF electrochemical sensor. (**A**) Schematic illustration of construction of the impedimetric aptasensor, and (**B**) Cascade catalysis amplification to form nonconductive insoluble precipitates (IPs). Reproduced from Ref. [41] with permission from Elsevier.

Liu and his coworkers successfully used a Ag-based MOF as a decoration of an electrode and showed that it provided stable electrochemical signals in a voltammetric technique alone. Using the Ag MOF-decorated electrode, it was able to detect CEA with a low LOD of 8.0 fg/mL. It should be mentioned that the voltametric immunoassay has been proven to be stable, inexpensive, sensitive, and selective [40]. Zhou et al., in 2017, fabricated a Cu MOF to be used as an electrochemical biosensor, where EIS measurements were used for detection [41]. The prepared Cu MOF nanostructures were further functionalized with Pt nanoparticles, aptamer, hemin, and glucose oxidase (Pt@CuMOFs-hGq-GOx). This biosensor assembly was found to mimic the peroxidase activity. Based on the cascade catalysis amplification driven by glucose oxidase, CEA was detected at a LOD of 0.023 pg/mL, as described in Figure 7 [41].

**Figure 8:**
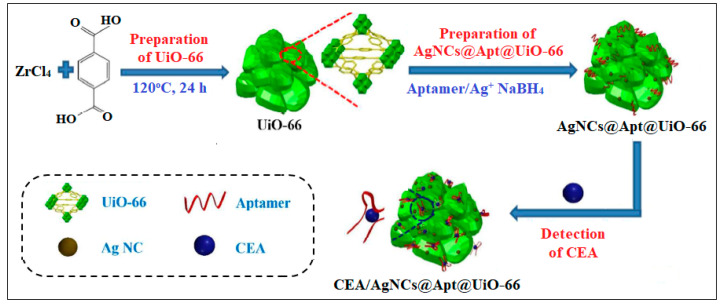
Schematic of silver nanocrystal with UiO-66 as a platform for CEA detection. Reproduced from Ref. [42] with permission from the American Chemical Society.

UiO-66, a well-known MOF, was also used in combination with Ag nanoclusters (Ag NCs) and CEA aptamer (AgNCs@Apt@UiO-66) for the detection of CEA [42]. The synthesized AgNCs@Apt@UiO-66 sensor was found to utilize both electrochemical and SPR techniques, as shown in Figure 8. Results showed that the proposed electrochemical AgNC@Apt@UiO-66-based aptasensor exhibits high sensitivity with a LOD of 8.88 and 4.93 pg/mL, as deduced from electrochemical impedance spectroscopy and differential pulse voltammetry, respectively. Meanwhile, the developed SPR biosensor exhibited a slightly high LOD of 0.3 ng/mL [42].

**Figure 9:**
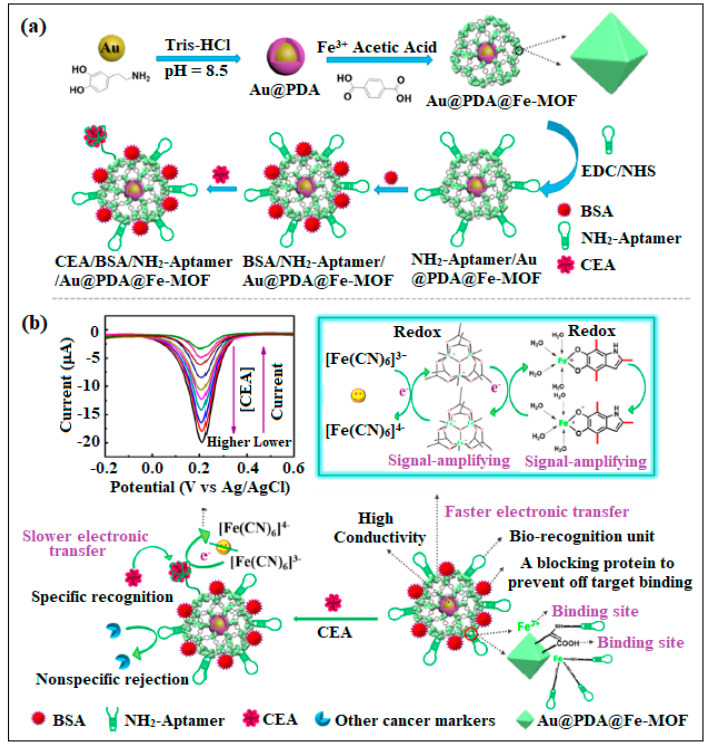
Schematic for the Fe MOF-based biosensor. (**a**) Schematic Representation for the synthesis Process, and (**b**) the Functioning Mechanism of Au@PDA@Fe-MOF and Electrochemical Aptasensor for CEA Detection.

In a novel approach, self-polymerized dopamine-decorated Au NPs that are also coordinated with Fe MOFs (Au@PDA@ Fe-MOF) was designed for the detection of CEA [43]. This nanocomposite was employed as a transducer and showed good electrochemical signals. In addition, this nanocomposite assembly was used to immobilize the recognition element (CEA aptamer) due to abundant COOH groups embedded in Fe MOFs, as shown in Figure 9. This sensor showed a high sensitivity and excellent selectivity [43].

**Figure 10:**
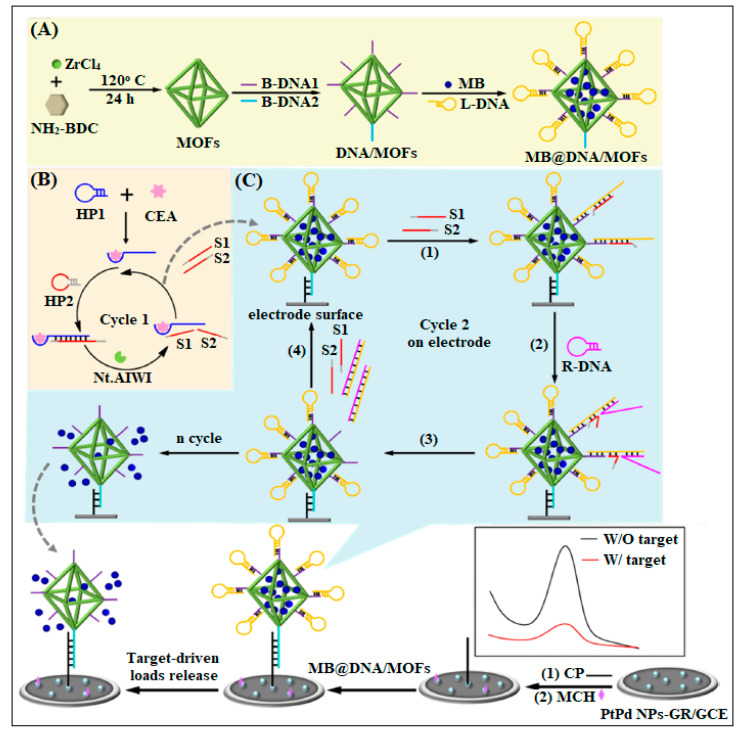
Schematic for the UiO-66 loaded with MB for electrochemical biosensing. (**A**) Scheme Diagram of the DNA-Gated MOF-Based Electrochemical Biosensing Platform of CEA shows the assembly of MB@DNA/MOFs. (**B**) Target-Triggered Nicking Endonuclease Cleavage Process. (**C**) Signal Molecule Release from MB@DNA/MOFs on the Electrode. Both aptamers S1 and S2 can recognized by L-DNA, and the exposed segment acted as a hold for R-DNA assembly. R-DNA was complementary with L-DNA to release S1 and S2 for recycling (cycle 2). With the cascade recycling happening, the capped L-DNA was detached from MB@DNA/MOFs, which made the unlocking of the pore and the release of MB, resulting in the reduction of electrochemical signal. Reproduced from Ref. [44] with permission from the American Chemical Society.

Bao et al. used UiO-66 as a nanocarrier of electroactive molecules (methylene blue, MB). DNA was further immobilized onto this composite (MB@DNA/MOFs), and was used as a platform for electrochemical biosensor, as described in Figure 10 [44]. The biosensor presented good performance for CEA detection ranging from 50 fg/mL to 10 ng/mL with a detection limit of 16 fg/mL [44]. In an earlier study, a type of dendritic DNA scaffold labeled with a Pb-based MOF, synthetized using a hybrid chain reaction as signal tags, was used for the detection of CEA. A detection limit of 0.333 pg/mL was obtained [45].

In a recent study by Zhang et al., both Au NPs and ordered mesoporous carbon (OMC) were incorporated with ZIF-8, and an electrochemical biosensor was prepared thereof [46]. Zif-8 served as a support nanocarrier for the immobilization and increased loading of the antibody onto its large surface area. The OMC was dropped on a glassy carbon electrode to improve electrochemical signals due to its intrinsic electrical conductivity. DPV was carried out to record the electrochemical responses. The sensor demonstrated excellent performance with a LOD of 1.3 pg/mL [46].

**Figure 11:**
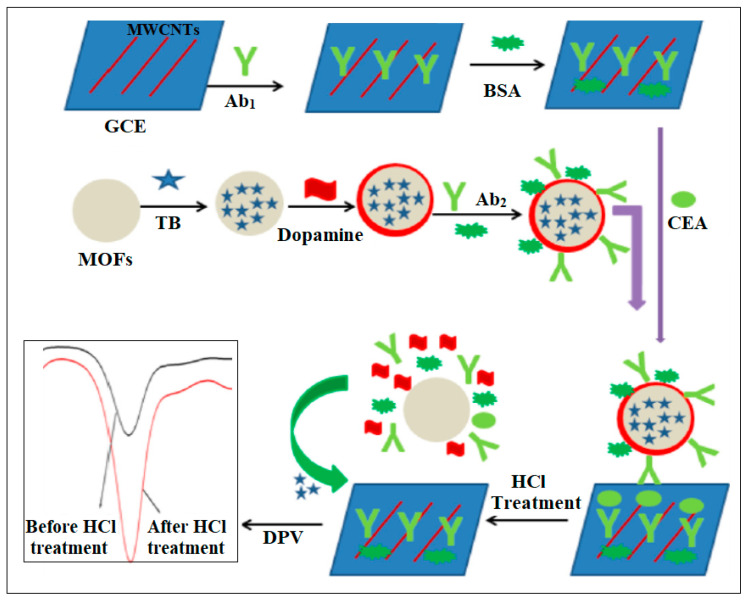
Schematic diagram for Cu MOF biosensor. Reproduced from Ref. [47] with permission from Elsevier.

Liu et al. revealed the synthesis of Cu MOFs by a hydrothermal method for establishing a sandwich-type sensor, as shown in Figure 11 [47]. Toluidine blue (TB)-loaded mesoporous Cu MOFs with PDA coating were employed as a signal transducer probe. The suggested sensor was fabricated by Cu MOFs-TB/PDA, chitosan, and multiwall carbon nanotubes (MWCNTs) on a glassy carbon electrode with a large surface area to immobilize primary antibodies (Ab1) [47]. Excellent conductivity was achieved, and CEA was quantitatively detected with LOD of 3.0 fg/mL under optimal conditions [47].

A platform for the ultrasensitive detection of three analytes: thrombin, kanamycin, and CEA, was also established by Zhang et al. [23] A series of Zr-based MOF composites embedded with three kinds of aptamer strands were achieved using a one-step de novo synthetic approach [23]. The label-free electrochemical aptasensors based on Zr MOF@Apt composites demonstrated high stability, repeatability, and applicability and showed excellent sensitivity to these analytes with detection limits of 0.40, 0.37, and 0.21 pg/mL for CEA, thrombin, and kanamycin, respectively [23].

In another study, three Sm-based MOF nanostructures were synthesized with different organic linkers, resulting in three different morphologies including rod-shaped, cubic consisting of stacked 2D layers, and spherical made of small cubic structures [48]. These Sm MOFs were used to fabricate immunosensors for the detection of CEA, where the CEA antibodies were immobilized onto the working electrode [48]. The exhibited reproducibility and selectivity of the immunosensors were proven with low limit of detection. They were tested with human serum samples, showing promising results [48].

### 2.2. Chemiluminescence Approach

Chemiluminescence (CL) has grown into a well-established luminometry method in analytical chemistry and has been widely applied to liquid-phase samples for over 30 years. CL is defined as the production of light through a chemical reaction that is accompanied by energy release. A CL biosensor is a type of analytical device that utilizes the emission of light from a chemical reaction to detect and quantify specific biological substances (analytes). It combines the principles of chemiluminescence, which is the production of light resulting from a chemical reaction, with the selectivity of biological recognition elements (e.g., enzymes, antibodies, DNA) to achieve sensitive and specific detection of target analytes [49].

It has numerous advantages such as superior sensitivity, rapidity, safety, and controllable emission rate. However, CL detection acquires extra facilities, such as online sample processing and inline multidetector installment [49]. For CL-based biosensors, the most utilized design is a flowthrough biosensor [50].

MOFs offer several advantages that make them well suited for chemiluminescence biosensors, including high surface area, tunable pore structures, stability, compatibility with diverse chemiluminescent probes, and biocompatibility [51].

A novel preparation of MIL-88B (Fe) using hemin and a metalloporphyrin Fe-based MOF, called as hemin@MIL-88B (Fe), was reported in 2020 by Han et al. [51] to be used for the CL biosensing of CEA. The hemin@MIL-88B (Fe) was conjugated with CEA aptamer1 (hemin@MIL-88B (Fe)-apt1) as the large signaling strategy. The ssDNA was immobilized on the surface of Fe_3_O_4_@SiO_2_ magnetic material, and hemin@MIL-88B (Fe)-apt1 was adsorbed on the Fe_3_O_4_@SiO_2_ by the complementary pairing of the partial bases between ssDNA and CEA apt1. The luminol CEA aptamer2 (L-apt2), which can generate the CL signal, was separately prepared, and was adsorbed on magnetic carbon nanotubes (M-CNTs) using electrostatic adsorption. Interestingly, hemin@MIL-88B (Fe)-apt1, L-apt2, and CEA formed in sandwich-like ternary complexes. The positively charged hemin@MIL-88B (Fe) easily accumulated near the hydroperoxide anion (HO^2−^) through electrostatic adsorption, thereby catalyzing the luminol transient chemiluminescence system. LOD was successfully reported to be 1.5 × 10^−3^ ng/mL [51].

### 2.3. Electrochemiluminescence Approach

**Figure 12:**
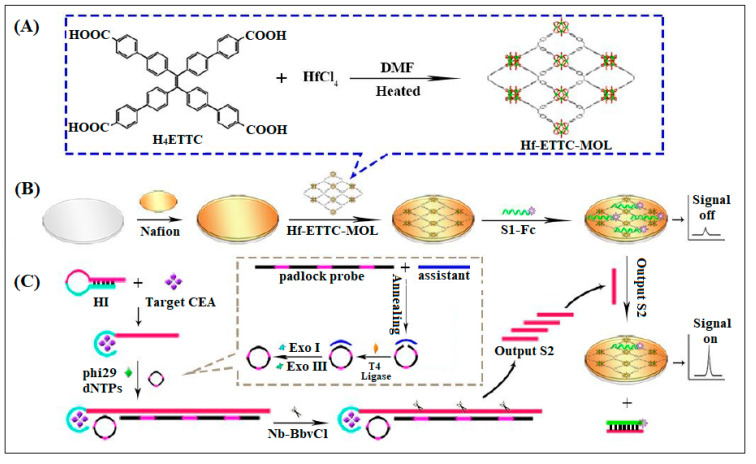
Schematic for MOL for CEA detection. (**A**) Formation of the ligand. (**B**) Electrode preparation by loading the MOF then the Ferrocene-labeled single-stranded DNA (Fc-S1) was introduced and strongly absorbed modified electrode. (**C**) Show the ECL “signal-off” state owing to the quenching effect of Fc. While in the presence of target CEA, hairpin probe (H1) recognized the target CEA and then a “signal-on” state. Reproduced from Ref. [54] with permission from the Royal Society of Chemistry.

On the other hand, electrochemiluminescence (ECL), which is defined as electrogenerated CL, possesses high sensitivity and broad dynamic range [52]. ECL differs from most other electrochemical techniques [52]. In fact, ECL relies on the generation of high energy electroactive species in the vicinity of electrode surface. As a result, ECL techniques have been widely used in immunoassays as a direct optical readout of electrochemical reactions. The ECL intensity of the targeted nanomaterials considerably depends on the corresponding electrocatalytic activity. In recent years, extensive research has been performed on ECL in combination with various nanomaterials, including MOFs. The brilliant features of MOFs make them promising candidates for ECL bioanalysis [53]. In a unique study, a Hf-based 2D MOF was fabricated and used in CEA detection, as described in Figure 12. The proposed 2D nanostructure exhibited higher ECL intensity and efficiency and was used to load an aptasensor for the ultrasensitive detection of CEA. A detection limit of 0.63 fg/mL was achieved. It should be mentioned that these findings provide strong new prospects for the development of novel ECL materials and will encourage more interest in the use of 2D MOF nanostructures for ECL sensing [54].

**Figure 13:**
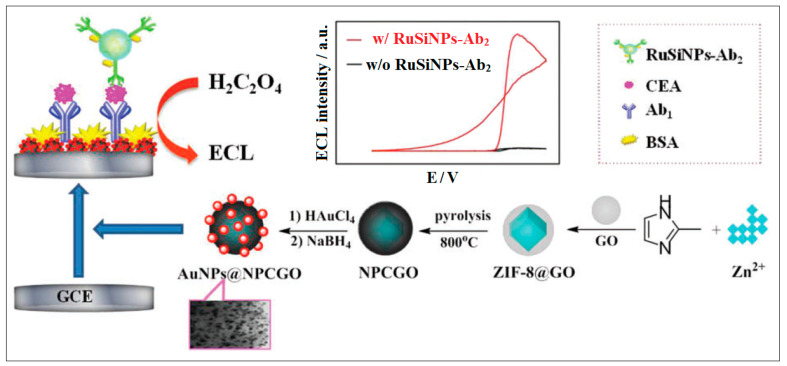
The procedure for preparing AuNP@NPCGO, the fabrication of the RuSiNP-Ab2 probe (Ab2 bioconjugates) and the stepwise preparation of the immunosensor. Reproduced from Ref. [55] with permission from the Royal Society of Chemistry.

Graphene oxide (GO)-doped ZIF-8 were also used for the fabrication of an ultrasensitive sandwich-type ECL immunosensor. The pyrolyzed nanocomposites were loaded with Au NPs, which were immobilized with antibodies. These nanocomposites were further loaded onto ruthenium (Ru)-labelled silica nanoparticles (RuSi NPs) and were used as ECL signaling units as they provided numerous luminophores that enhanced the ECL, as shown in Figure 13 [55].

**Figure 14:**
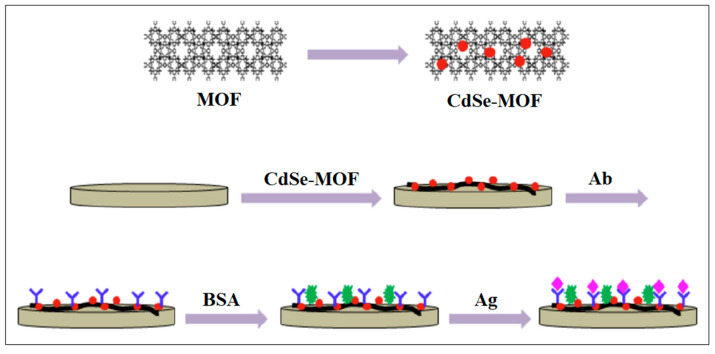
Electrochemiluminescence MOF-based biosensors were successfully synthesized by combining CdSe QDs and MIL-101. Reproduced from Ref. [56] with permission from Elsevier.

Through the combination of MIL-101 and CdSe quantum dots (QDs), an ECL sensor was successfully synthesized, as described in Figure 14. The as-prepared nanocomposites were applied to immobilize the antibody of the CEA, and a high ECL activity and sensing selectivity were obtained, where a detection limit of 0.33 fg/mL was observed [56].

### 2.4. Fluorescence Approach

Fluorescence is by far the most commonly used method in the design of biosensors, as described in many studies. Peculiarly, fluorescent MOFs have been used in in these research directions. The fluorescence properties of MOFs are generated by metal ions and organic ligands. Moreover, other fluorescent molecules are encapsulated within the MOF channels and can produce emission fluorescence [57]. These characteristics make MOF materials suitable candidates for the fabrication of MOF-based fluorescence sensors for specific targeted species such as metal ions, anions, and small molecules [58].

**Figure 15:**
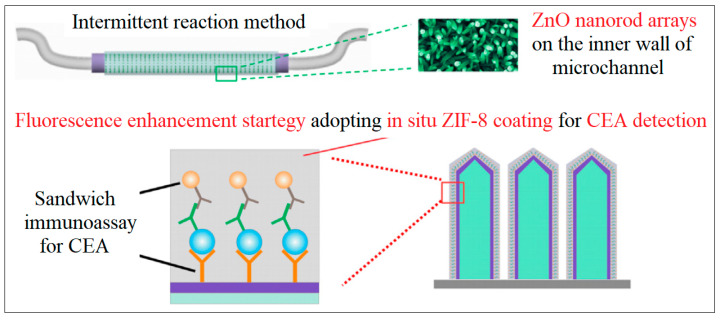
Microfluidic channels for fluorescence detection of CEA. Reproduced from Ref. [59] with permission from Elsevier.

A promising fluorescence-based microfluidic sensor for the quantification of CEA was developed by Zhao et al., as schematically represented in Figure 15 [59]. In this work, a synergetic fluorescence enhancement strategy based on micro/nanostructure optimization of ZnO nanorod arrays and in situ ZIF-8 coating was proposed. A glass capillary was chosen as the microfluidic channel, and controllable construction of ZnO nanorod arrays on the inner wall of the microchannel was conducted via the intermittent reaction method. The fluorescence enhancement characteristics of ZIF-8 towards organic fluorescence labels were investigated and successfully applied to protein marker detection. The LOD reached was as low as 0.01 pg/mL [59].

In a different study, an innovative and powerful visible fluorescence immunoassay method was fabricated through a wet NH_3_–triggered structural change of NH_2_-MIL-125(Ti) impregnated on paper for the detection of CEA [60]. Au NPs heavily functionalized with glutamate dehydrogenase and secondary antibody were used for the generation of wet NH_3_ in a sandwiched immunoassay format. A paper-based analytical device (PAD) coated with NH_2_-MIL-125(Ti) exhibited good visible fluorescence intensity through wet NH_3_-triggeried structural change with high accuracy and reproducibility. Moreover, the NH_2_-MIL-125(Ti)-based PAD displayed two visual modes of fluorescence color and physical color to the naked eye and allowed for the detection of CEA at a concentration of as low as 0.041 ng/mL. Importantly, the PAD-based assay provides a promise for the mass production of miniaturized devices and opens new opportunities for protein diagnostics and biosecurity [60].

### 2.5. Photoelectrochemical Approach

The photoelectrochemical (PEC) process is a promising low-cost approach to converting chemical energy to electricity under light illumination and applied potential. PEC biosensing has attracted huge attention because of its ability to detect biomolecules through the photocurrent generated from a biomolecule’s oxidation. The ability to couple the photoexcitation process with electrochemical detection renders PEC sensors unique. Moreover, the separation between the sources of excitation (light) and detection (photocurrent) in the PEC process offers high sensitivity with low background signal [61].

**Figure 16:**
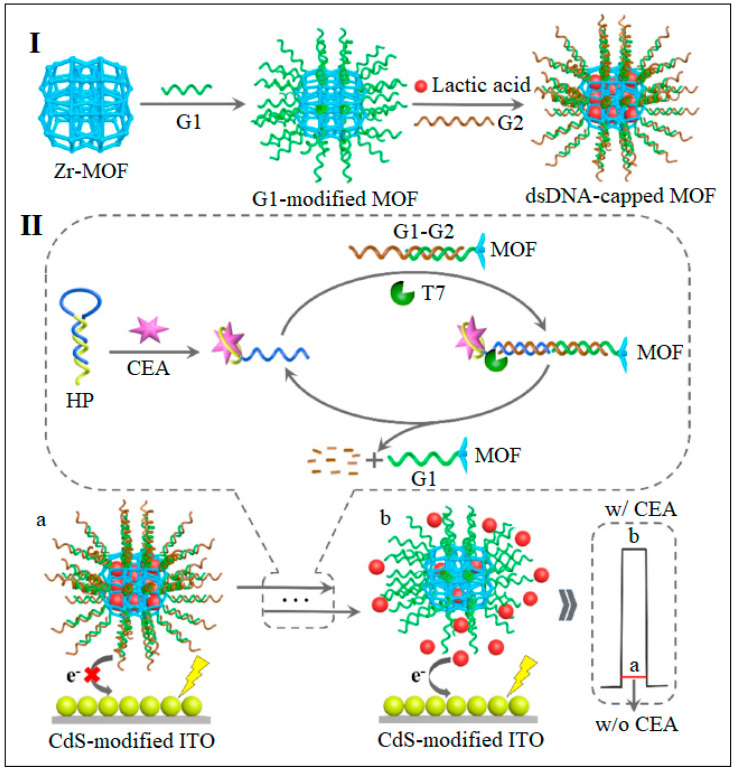
Zr MOF (UiO-66) as a carrier for electron donor. (**I**) Schematic for the fabrication of biosensor fabrication and surround it with ssDNA G1, G2. (**II**) Shows the sequence of the ssDNA region in G1–G2 was complementary to part of the hairpin probe (HP). HP contained a specific aptamer sequence for CEA. In the presence of CEA, the combination of its aptamer sequence and CEA triggered the conformational change of HP, which could hybridize with the protruding ssDNA region in G1–G2 and procedure a blunt duplex conformation. This duplex could be detected by T7 exonuclease, which digest the G2 and the release of CEA–HP complex, Reproduced from Ref. [62] with permission from Elsevier.

Liu et al. proposed a photoelectrochemical biosensor based on a UiO-66 MOF [62]. This work established an immobilization-free PEC biosensor based on the DNA-functionalized MOF. Here, UiO-66 served as a nanocarrier for the efficient encapsulation of electron donors, while a designed probe was employed as the recognition element (Figure 16). The proposed biosensor was found capable of ultrasensitive and highly selective determination of CEA with a detection limit down to 0.36 fg/mL [62]. CEA assay could be realized by comparing the change of the photocurrent signals, when the CEA present a strong photocurrent can be recognized while in the absence of CEA no photocurrent is produced.

A recent PEC immunosensor based on a Yb MOF was designed for the high performance determination of CEA [63]. The surface of the Yb MOF was integrated with AuNPs to improve the photoelectric conversion efficiency of the Yb MOF in the near-infrared region. Subsequently, this nanocomposite became a photoelectrochemical platform for loading the CEA antibody (anti-CEA). After exposing it to CEA, the photogenerated electron–hole pair transfer was blocked thus leading to a decrease in photocurrent response. The photocurrent variation can be used for determining CEA quantitatively. The range of detection was measured from 0.005 to 15 ng/mL [63].

### 2.6. Colorimetric Approach

Colorimetric sensors and biosensors exhibit promising potential due to their simplicity and reliability as potential low-cost platforms. The main prospect of colorimetric sensors is based on the interaction between light and metal nanomaterials. In particular, the color is created by the change in absorbance due to the optical properties of the material. The change in absorbance can be measured as a function of the different concentrations of clinical biomarkers [64]. For CEA colorimetric detection, a group of researchers synthetized a novel biosensor based on a PCN-222 MOF, which was prepared by grafting CEA aptamer-incorporated DNA tetrahedral (TDN) nanostructures [65]. The synthesized CEA aptamer-TDN-MOF showed a high detection stability due to the iron porphyrin ring in the PCN-222 MOF, which possesses significant horseradish peroxidase-mimicking activity, which leads to a colorimetric reaction upon binding toward antibody-captured CEA. Ultrasensitive detection of CEA can be achieved with a limit of detection as low as 3.3 pg/mL. In addition, they demonstrated a great potential upon the analysis of clinical serum samples [65]. In a latest study, Zeng and his colleagues designed and fabricated a colorimetric immunosensor by taking advantage of 2D MOF nanomaterials as enzyme mimics [66]. The nanomaterial showed strong peroxidase mimetic activity and good selectivity after being modified with a specific aptamer. They reported a CEA detection performance with a linear range from 1 pg/mL to 1000 ng/mL and LOD of 0.742 pg/mL [66].

ELISA has been identified as a gold standard and is the most widely used immunoassay technique for detecting and measuring disease biomarkers. However, there are some inevitable limitations in the use of ELISA, including limited sensitivity, which requires novel strategies to improve the ELISA limit of detection. For the enhancement of ELISA, researchers introduced MOFs during the fabrication of an ELISA platform. The MOF-based ELISA provides large surface-to-volume ratios, which promote the efficient immobilization of antibodies on the nanoscale surface that consequently increase the capture efficiency of substrate surfaces.

**Figure 17:**
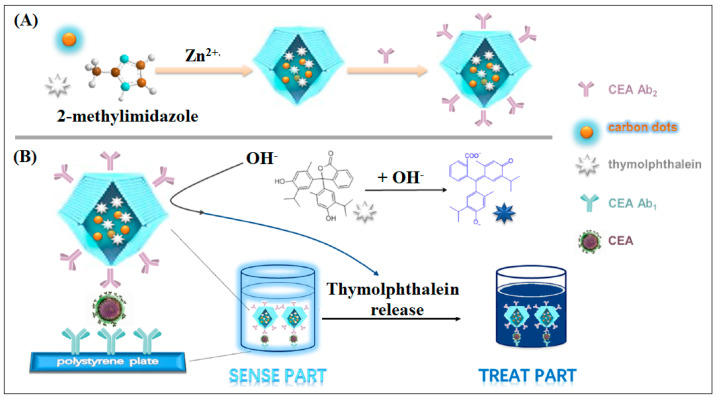
ZIF-8 For ELIZA detection of CEA. (**A**) is the synthesis step of the MOF contain Quantum dots and thymolphthalein and surrounded with Abs, (**B**) Schematic representation for the second step illustrating the CEA detection. Reproduced from Reference [67] with permission from Elsevier.

In one of the most interesting studies, ZIF-8 doped with carbon dots (CDs) and thymolphthalein was used as a platform for CEA detection via ELISA technique, as shown in Figure 17. In this nanocomposite sensor, the strong fluorescence intensity of CDs could be observed directly to achieve the sensitive detection of the target. After stimulation of alkaline solution, TP was released from ZIF-8 carriers and generated a color change with an obvious absorption, which was beneficial to the increased sensitivity of this ELISA due to the high loading of thymolphthalein [67].

More details on the different types of MOFs used for the development of biosensors for the detection of the CEA biomarker are provided in Table 1. Moreover, the method of structure modification, type of sensing, and limits of detection are provided.

## 3. Conclusions and Future Perspectives

As one of the common tumor markers, CEA has important clinical values in the differential diagnosis, disease monitoring, and therapeutic evaluation of malignant tumors. In the past few decades, advances in biosensor technology have led to the evolution of simple, fast, low-cost detection, high sensitivity, and good selectivity biosensors for various diseases. Compared with other nanostructures, MOFs provide high surface area, high functionality, tunable porosity, non-expansive, and versatile fabrication procedures. These features have made them appropriate choices for the establishment of a wide variety of CEA sensors. Generally, this minireview thoroughly discussed the most recent advances in the design and fabrication of MOF-based biosensors for the detection of the CEA cancer biomarker. The optical and electrochemical properties of MOF nanostructures have shown an outstanding ability to detect analytes at low concentrations. Moreover, these can be further enhanced through the development of hybrid nanostructures via the inclusion of other nanomaterials. For example, using luminescent scaffolds such as QDs or fluorescent dyes would result in the development of more accurate and sensitive optical devices. On the other hand, the integration of MOFs with conductive polymers would significantly enhance electrochemical sensing characteristics.

Despite the pronounced features of MOFs as a core material for the development of biosensors, more research is still needed to tackle the arising limitations of MOF nanostructures, such as their stability, reproducibility, toxicity, and biocompatibility. One of the primary restraints is their low water stability, which tends to cause the breakdown of the framework when exposed to moisture. In addition, more attention should be paid to the validation of MOF-based biosensors in the analysis of real biological samples, with a more emphasis afforded to any possible interferences that may affect the sensing results. Addressing these issues is crucial before the implementation and commercialization of MOF-based biosensors.

## Figures and Tables

**Table 1:** MOF-based CEA biosensors.

#	MOFs	Metal Used	Organic Ligand	Surface Modifications and Added Materials	Types of Sensing	LOD/Detection Range	Reference
1	Pd/Cd MOFs	Pd/Cd	2-aminoterephthalic acid	Immobilization of labels for secondary anti-CEA and secondary anti-AFP antibody	Electrochemical	0.03 pg/mL and 0.1 pg/mL	[38]
2	Ce MOF	Ce	1,3,5-benzenetricarboxylic acid	Hyaluronic acid was coated on the surface of a Ce MoF that was loaded with silver nanoparticles (AgNPs) and horseradish peroxidase	Electrochemical	0.2 pg/mL	[39]
3	Ag MOF	Ag	Terephthalic acid	A Ag MOF was dopped using gold nanoparticles and labelled with anti-CEA	Electrochemical	8.0 fg/mL	[40]
4	Cu MOF	Cu	2-amino terephthalic acid	Platinum nanoparticles (PtNPs) were linked to a Cu MOF; then, a CEA aptamer was loaded onto Pt@CuMOFs; finally, this was bound with hemin to form hemin@G-quadruplex (hGq) with mimicking peroxidase activity	Electrochemical	0.023 pg/mL	[41]
5	UiO-66	Zr	2-Aminoterephthalic acid	MOF embedded with silver nanoclusters (AgNCs) using the carcinoembryonic antigen (CEA)-targeted aptamer as template	A. Electrochemical 1—Impedance	8.88 pg/mL	[42]
					2—Differential pulse voltammetry	4.93 pg/mL	
					B. SPR	0.3 ng/mL	
6	Fe MOF	Fe	1,4-dicarboxybenzene	Self-polymerized dopamine-decorated AuNPs were loaded on an Fe MOF and attached to a CEA aptamer	Electrochemical	0.33 fg /mL	[43]
7	UiO-66-NH2	Zr	2-Aminoterephthalic acid	By using MOF as nanocarrier of electroactive molecules (methylene blue, MB) and functionalized by the assembled DNA	Electrochemical	16 fg/mL	[44]
8	Pd MOF	Pd	2-amino-1,4-benzenedicarboxylic acid (H_2_N-BDC)	Dendritic (hybridization chain reaction) HCR-triggered DNA nanostructure was labeled with Pb MOF	Electrochemical	0.333 pg /mL	[45]
9	Zif-8	Zn	Methyl Imidazole	It is based on the use of a Au NP-modified ZIF-8 and ordered mesoporous carbon (OMC)	Electrochemical	1.3 pg/mL	[46]
10	Cu MOF	Cu	Terephthalic acid	Toluidine blue (TB) loaded mesoporous Cu MOFs with polydopamine (PDA) coating were employed as a signal probe	Electrochemical	3.0 fg/mL	[47]
11	Zr MOF	Zr	4′,4‴,4′′′′-nitrilotris [1,1′-biphenyl]-4-carboxylic acid (H3NBB)	Aptamers of CEA, thrombin, and kanamycin were separately immobilized on the MOF	Electrochemical	0.40 pg/mL 0.21 pg/mL 0.37 pg/mL	[23]
12	Sm MOF	Sm	Trimesic acid (TMA), meso-tetra(4-carboxyphenyl)porphine (TCPP), and 1,3,6,8-tetra(4-carboxylphenyl) pyrene(TBPy)	Anti-CEA immobilization	Electrochemical	SmTMA, SmTBPy, and SmTCPP MOF-based immunosensors are determined to be 0.001, 0.05, and 0.01 U/mL, respectively	[48]
13	MIL-88B	Fe	2-aminoterephthalic acid	Hemin-modified Mil-88B Immobilization of CEA aptamer	CL	1.5 × 10^−3^ ng/mL	[51]
14	Hf-ETTC-MOL	Hf	H_4_ETTC (H4ETTC = 4′,4′′′,4′′′′′,4′′′′′′′-(ethene-1,1,2,2-tetrayl)tetrakis(([1,1′-biphenyl]-4-carboxylic acid)))	A two-dimensional (2D) ultrathin metal–organic layer (MOL) was used as a platform for CEA detection	ECL	0.63 fg/mL	[54]
15	Zif-8	Zn	Methyl imidazole	ZIF-8 and graphene oxide (GO) to form a ZIF-8@GO composite. Then, the in situ growth of AuNPs due to the p–p interaction between AuNPs and Zif-8@GO took place	ECL	0.003 ng/mL	[55]
16	MIL-101	Cr	Terephthalic acid	Prepared MIL-101-CdSe nanocomposites antibodies for CEA was linked	ECL	0.33 fg/mL	[56]
17	Zif-8	Zn	Methyl Imidazole	Formation of sandwich immunoassay by using Zif-8 coated with ZnO/PAA (polyacrylic acid) nanorod arrays	Fluorescence	0.01 pg/mL	[59]
18	NH_2_-MIL-125(Ti)	Ti	2- amino-1,4-benzenedicarboxylic acid (H_2_N-BDC)	Gold nanoparticles heavily functionalized with glutamate dehydrogenase (GDH) and secondary antibody were used for generation of wet NH_3_	Fluorescence	0.041 ng/mL	[60]
19	UiO-66-NH_2_	Zr	2-Aminoterephthalic acid	MOFs loaded with lactic acid and attached to dsDNA	PEC	0.36 fg/mL	[62]
20	Yb MOF	Yb	1,1′-(1,5-dihydropyrene-2,7-diyl)bis(3-(4-carboxybenzyl)-1H-imidazol-3-ium) bromide [DDPDBCBIm(Br)2] ionic liquid	Combined with gold nanoparticles	PEC	0.005–15 ng/mL	[63]
ch21	PCN-222	Zr- and Fe-based MOF	meso-tetra (4-carboxyphenyl) porphine ferric chloride (Fe-TCPP)	A CEA aptamer was immobilized on PCN-222	Colorimetric	3.3 pg/mL	[65]
22	Cu TCPP	Cu-MOF	TCPP	Gold nanoparticle immobilization and aptamer	Colorimetric	1 pg/mL to 1000 ng/mL	[66]
23	Zif-8	Zn	Methyl Imidazole	ZIF-8 used as the carrier to deliver the tracer agent carbon dots (CDs) and the “drug” thymolphthalein (TP)	ELIZA	10 pg/mL	[67]

## Data Availability

This is a review article, where data mentioned are available on the cited work of other colleagues in their respective article.

## Abbreviations

CEA	Carcinoembryonic antigen
MOFs	Metal–organic frameworks
ELISA	Enzyme-linked immunosorbent assays
RIA	Radioimmunoassay
IRMA	Immunoradiometric assay
RT-PCR	Reverse transcriptase polymerase chain reaction
MIPs	Molecular imprinted polymers
AuNPs	Gold nanoparticles
CNT	Carbon nanotubes
CV	Cyclic voltammetry
DPV	Differential pulse voltammetry
SWV	Square wave voltammetry
EIS	Electrochemical impedance spectroscopy
GCE	Glass carbon electrode
AFP	Alpha fetoprotein
AgNPs	Silver nanoparticles
LOD	Limit of detection
PDA	Polydopamine
OMC	Ordered mesoporous carbon
MWCNTs	Multiwall carbon nanotubes
CL	Chemiluminescence
ECL	Electrochemiluminescence
GO	Graphene oxide
QDs	Quantum dots
PAD	Paper-based analytical device
PEC	Photoelectrochemical
TDN	DNA tetrahedral
CDs	Carbon dots
SRP	Surface Plasmonic Resonance
